# Poor-Law Infirmaries and the War Office

**Published:** 1914-08-29

**Authors:** 


### Poor-Law Infirmaries and the War Office.

### BIRKENHEAD.

*(From our Special Correspondent.)*

The Birkenhead Guardians have been engaged for some years in reconstructing the Birkenhead Union Infirmary. It is now finished, and has been held up to the Brownlow Hill authorities by the Local Government Board as a pattern. Ample provision having been made for years to come, the Guardians have offered the war authorities 100 beds *separate* from other patients, with a possible fifty more, also *separate*, in stress. Besides this, they have consented to take such cases as may not be able to be sent home from the Borough (Voluntary) Hospital, so that that body can provide some wards for the war authorities if needed. The Borough Hospital does very useful accident and emergency work, but has only seventy-two beds all told. It may also be mentioned that the Birkenhead Union Infirmary is separately administered from the workhouse by Order April 1, 1913.

### OFFERS OF POOR-LAW INSTITUTIONS.

Since our last issue many more Poor-law Guardians have offered vacant accommodation in their institutions to the War Office. The Fulham Guardians have offered 100 beds in their infirmary, Tendring (Essex) 50 beds, Sunderland the whole of their recent infirmary extensions (46 beds), North Bierley (Yorkshire) any vacant accommodation available, the Loddon and Clavering Union (Norfolk) 40 beds, Dunmow 20 beds in an empty block of the workhouse, and the Thornbury Union (Gloucestershire) have placed the whole of their infirmary wards at the disposal of the Red Cross Society. Many infirmaries have also arranged to admit Red Cross Society nurses, so that they may obtain training and experience in the nursing of the sick. The Lambeth Guardians have arranged to receive into their infirmary patients displaced from St. Thomas's Hospital owing to the requirements of the naval and military authorities.

### SOUTH SHIELDS.

*(From our Special Correspondent.)*

The Harton Hospital, South Shields Union, has cleared sixty beds, and these are ready for immediate occupation. Dressings and surgical appliances are prepared and ready for use. This hospital is equipped with a central kitchen for hospital cooking entirely. There is an operating theatre and an $x$-ray installation. In case of real emergency the accommodation can be doubled by using certain of the workhouse dormitories as temporary wards for suitable hospital patients. The whole of the wounded would thus be dealt with in the hospital building itself. Dr. Bootiman, of South Shields, is Admiralty Agent for the district, and it is he who is organising the accommodation in the town. Further particulars as regards this infirmary are that Dr. Bootiman has arranged for three medical men, Dr. Bootiman, Dr. Dalziel, and Dr. Orde, to attend there, and together with Dr. J. Dudgeon Giles, the medical superintendent and his assistant medical officer, to attend to the cases sent. Three or four horse ambulances (two of them from Harton Hospital) and about twelve hand ambulances are available for the transport of the wounded from Tyne Dock to the hospitals.

The Guardians' new motor ambulance, of which they shortly expect delivery, will also be available. The Ingham Infirmary is able to place sixty beds at the service of the authorities, and a temporary hospital is established in St. Bede's School. Additional nursing assistance would also be available if a rush of cases came in. The Admiralty surgeon who visited this hospital a few days ago to inspect its preparations has expressed himself as very well satisfied.

Dr. J. Robertson Crease has been busy successfully converting buildings and schools into fully-equipped hospitals, properly staffed, with a small army of messenger boys and stretcher bearers ready to bring in and attend to any wounded. There are three hospitals working under his direction.

[Brief reference to the offers of other Poor-Law infirmary authorities is made in our Notes this week. See "The Combined War Scheme at Bristol."]

### An East Coast Town's Preparations.

Preparations are now practically complete for dealing with any wounded who may arrive in Sunderland. Situated as it is on the North-East

Coast, the port would be one of the centres to which hospital ships would come should an encounter take place in any part of the North Sea between Norway and Scotland. Arrangements have been made for landing the men at certain quays alongside which are railway lines. It is expected that the authorities would be able to give something like an hour's notice of a ship coming in. When she arrives trains will be in readiness along the quay, and in these the more serious cases will be placed and conveyed as speedily as possible to the Royal Infirmary. Bearers will also be in attendance with ambulances and motors to convey the minor cases to various public institutions and temporary hospitals. Everything is in readiness to meet the call whenever it comes, day or night. The whole of the local arrangements have been inspected by Fleet-Surgeon Wilson, of London, accompanied by Dr. Gordon Bell, local Admiralty Medical Officer. The Sunderland Royal Infirmary is placing 150 beds at the disposal of the authorities—140 for men and ten for officers—whilst the Children's Hospital in connection with the Royal Infirmary is fitting up a ward of twenty beds. Seven nurses have also left the former institution for military service. In addition to the above, beds are also being provided at the Sunderland and Durham County Eye Infirmary, the Monkwearmouth and Southwick Hospital, and at the Union Hospital, and the 6th and 12th Volunteer Detachments have fully equipped several temporary hospitals in various parts of the town, which, should the emergency arise, will relieve the strain on the public institutions. The chemists of Sunderland have decided to make a present of the initial chemical stores and dressings for the temporary hospitals.

